# Book Reviews

**Published:** 1889-05

**Authors:** 


### BOOK REVIEWS.

Wood's Medical and Surgical Monographs. Volume one, numbers one and two, January and February.

The first number for January contains

*The Pedigree of Disease*, by Jonathan Hutchinson, F.R.S.;

*Common Diseases of the Skin*, by Robert M. Simon, M.D.;

*Varieties and Treatment of Bronchitis*, by Dr. Ferrand.

The second number, for February, contains

*Gonorrhœal Infection in Women*, by William Jap Sinclair, M.A., M.D.;

*On Giddiness*, by Thomas Grainger Stewart, M.D.;

*Albuminuria in Bright's Disease*, by Dr. Pierre Jeanton.

The subjects chosen for these two numbers are interesting, and the names of some of the authors are so well and so favorably known that one always feels assured that what comes from their pens will be worth reading.

The plan of the publishers, in issuing these shorter works in this form, gives to the subscriber more of a variety in the works furnished during a year than the plan followed in their *Library of Standard Medical Authors*, and at less expense to the purchasers, notwithstanding the difference in the binding. In these days when the profession is flooded with medical literature ranging through a scale all the way from good to bad—with many different shades between those two extremes—it is some relief to the reader to have some one make at least a partial selection for him, and these monographs will, to a certain extent, serve that purpose.

With exception of the binding, these two numbers have been issued in better shape than were the numbers in the preceding years covering the issue of the Standard Medical Authors.

American Resorts, with Notes upon Their Climate. By Bushrod W. James, A.M., M.D., Member of the American Association for the Advancement of Science; The American Public Health Association, etc., etc. Intended for invalids and those who desire to preserve good health in a suitable climate. Philadelphia: F. A. Davis. 1889. Octavo. 300 pages, cloth.

This work is divided into twelve chapters as follows:

Medical Climatology.
Benefits and Dangers of Health Resorts.
Sea-side Resorts.
Fresh-water Resorts.
Mountain Resorts.
Trips upon Ocean, Lake and River.
Mineral Springs.
Summer Resorts.
Winter Resorts.
Therapeutics.
Mexico and South America.
Translation from "Die Klimate der Erde."

These are followed by extensive bibliography of the subject, and accompanied by a map of both North America and Central America.

The work contains much information on the subject, which will interest those for whose benefit it was especially prepared. If it shall have, as one effect, an influence in awakening some of the nomadic Americans to the fact of the many advantages which their own country offers to seekers after health and amusement, the book will not have been written in vain. Certainly nature has not only been kind to us but lavish, and if art will but contribute its fair share in embellishing these naturally attractive resorts, and surround more of them with the comforts which modern civilization demands, the annual exodus of Americans to the older and better-known foreign resorts will probably be materially diminished in the near future.

Atlas of Venereal and Skin Diseases, comprising Original Illustrations and Selections from the Plates of Prof. M. Kaposi, etc., with Original Text. By Prince A. Morrow, A.M., M.D., Clinical Professor of Venereal Diseases, etc. New York: Wm. Wood and Co. Fasciculi, Nos. x. xi. and xii.

The last published fasciculi of this valuable and important collection of plates include admirable portraits of such common and rare affections as herpes in all varieties, many of the forms of dermatitis, pemphigus, purpura, eczema, molluscum, acne, and lichen planus. It is difficult to overestimate, from the point of view of the diagnostician, the value of this Atlas. If it had been made up entirely from the cases collected for illustration from the experience of one man, or of a body of men living in one country, it could not possess the worth it now has, representing as it does a truly catholic review of what has been hitherto published in all countries and places. Nor need Americans feel slighted of representation in so goodly a company of medical artists, for not least among the valuable lithographs in color here given are the author's own illustrations of disease. The text is a trustworthy commentary on the subjects presented in the portraits, and is filled with practical hints. The Atlas may be warmly commended to both students and practitioners as an invaluable addition to a medical library.

Handbook of the Diagnosis and Treatment of Skin Diseases. By Arthur Van Harlingen, M.D., Professor of Diseases of the Skin in the Philadelphia Polyclinic, etc. Second Edition. Enlarged and Revised. Philadelphia: P. Blakiston, Son and Co. 1889. Pp. 410.

The second edition of this valuable little work includes a number of new articles relating chiefly to the rarer affections of the integument; and also numerous original and selected illustrations which greatly enrich its pages. Many of the older chapters bear unmistakable evidences of careful revision. The first edition of the work greatly commended itself to the student and practitioner by reason of the exceedingly practical character of its recommendations for treatment: and it has evidently been the author's aim to fully sustain this reputation in the quality of its second edition. The book may be warmly commended to those who have need of a compendious hand-book of cutaneous medicine: and is easily first of the smaller and less pretentious of these works. Its author is well known as a competent and judicious contributor to the dermatological literature of this country. To those who have had a practical acquaintance with the pages of the first edition, it will be superfluous to commend this its successor, which has all the merits of the first and the advantages over it of a conscientious revision and enrichment.

The Psychic Life of Micro-Organisms.—A Study in Experimental Psychology. By Alfred Binet. Translated from the French by Thomas McCormack, with a preface by the author written especially for the American edition. Chicago: 1889. The Open Court Publishing Company. Cloth, 75 cents. Paper, 50 cents:

M. Alfred Binet, the collaborator of Ribot and Féré, and one of the most eminent representatives of the French School of Psychology, has presented in the above work the most important results of recent investigations into the world of Micro-Organisms. The subject is a branch of comparative psychology little known, as the data of this department of natural science lie scattered for the most part in isolated reports and publications, and no attempt has hitherto been made to collate and present them in a systematized form.

The cuts, eighteen in number, are illustrative of the movements, nutrition, digestion, nuclear phenomena, and fecundation of Proto-Organisms.

The most interesting chapters are those on fecundation, which demonstrate the same instincts and vital powers to exist in spermatozoids as are found in animals of higher organization.

A Manual of Instruction in the Principles of Prompt Aid to the Injured, Designed for Military and Civil Use. By Alvah H. Doty, M.D., Major and Surgeon, Ninth Regiment N. S. G. N. Y.: Attending Surgeon to Bellevue Hospital Dispensary, New York. New York: D. Appleton and Company. Chicago: A. C. McClurg and Company. Pp. xxiii. and 224.

The design of this little manual is to instruct those who are desirous of knowing what course to pursue in emergencies, in order that the sick or injured may be temporarily relieved, and the design has been well carried out. Like other books of the kind, it would serve a good purpose if more of those for whom the work was prepared would profit by its teachings—and early enough to know, when the emergency arises, what should be done, before proper medical or surgical aid can be obtained. It is seldom that non-professional persons acquaint themselves sufficiently with the requirements of such emergencies, before their occurrence, and for this reason many of the efforts made to so enlighten them that they can act intelligently and promptly fail to accomplish that object.

This work has been carefully prepared, and it is exceptionally well illustrated.

The Pathology, Clinical History and Diagnosis of Affections of the Mediastinum, other than those of the Heart and Aorta, with tables giving the clinical history of five hundred and twenty cases. Being an Essay to which was awarded the Fothergillian Medal of the Medical Society of London, March, 1888. By Hobart Amory Hare, B. Sc., M.D., etc., etc. Pp. 150. 1889. Philadelphia: P. Blakiston, Son & Co. Chicago: W. T. Keener.

Prior to the preparation of this essay, it is stated, but fifty-three references of cases of affections of the mediastinum had been collected by any one person, which is a surprising statement, especially when it is realized that the author of this essay has collected almost ten times that number, including:

| | | | |
|---|---|---|---|
| 134 | Cases of | Mediastinal cancer. | |
| 98 | " " | " sarcoma. | |
| 115 | " " | " abscess. | |
| 16 | " " | non-suppurative inflammation. | |
| 21 | " " | Lymphoma of the mediastinum, | |
| 7 | " " | Fibroma " " | |
| 6 | " " | Hæmatoma " " | |
| 11 | " " | Dermoid cyst " " | |
| 8 | " " | Hydatid " " " | and |
| 104 | " " | various Mediastinal diseases. | |

After giving tables classifying these different affections, and giving their clinical history, a brief summary of conclusions is given, as follows:

1. Cancer is more frequently found in the mediastinal spaces than any other morbid process.
2. Abscess is the morbid process next in frequency of occurrence.
3. Sarcoma occupies a third position as to frequency.
4. Lymphomata and Lymphadenomata occupy a fourth place, but are much more rare than the others mentioned.
5. The Anterior Mediastinum is affected far more frequently than the other two spaces.
6. Most Mediastinal growths occur in adults.
7. More males are affected than females by mediastinal disease, be that disease what it may.
8. Cancer and sarcoma of this space are necessarily fatal.
9. Abscess is recovered from in about 40 per cent. of the cases.

Six plates accompany the essay, giving views of some of these pathological conditions.

The result of the essayist's labor will be to make the profession more familiar with this subject, and to appreciate more fully how little attention it has heretofore received.

Transactions of The American Surgical Association. Volume Sixth. Edited by J. Ewing Mears, M.D., recorder of the Association. Printed for the Association and for sale by P. Blakiston, Son & Co., Philadelphia. Pp. xxvii and 572. 1888.

The volume embraces a list of the officers of the Association for the year; a list of the Presidents of the Association from the time of its institution; the names, and official positions, of the Fellows of the Association, and Honorary Fellows, and a necrological report for the year.

The scientific work of the Association, which embraces so many of the representative general surgeons of the United States, is such as would be expected from the active workers in the profession. Many of the papers have already been presented so fully to the profession, since the last meeting of the Association, as to be quite familiar, and not to call for any extended notice of them here.

A Compendium of Dentistry, for the use of Students and Practitioners. By Jul. Parreidt, Dental Surgeon to the Surgical Clinic of The Institute of the Unversity of Leipzig, etc. Authorized translation by Louis Ottofy, D.D.S., etc., etc. With Notes and Additions by G. V. Black, M.D., D.D.S., etc., etc. With numerous Illustrations. Pp. ix and 229. Chicago: W. T. Keener.

Whilst this work is especially interesting to dentists, it is not wantiug in interest or usefulness to physicians. Aside from the beauty of good teeth, and their great value in distinct articulation, the important part which they perform in the complicated process of digestion has had too little practical recognition. The simple mechanical work of the dentist has too often been given such prominence as to have led to the overlooking of the importance anatomically, physiologically and pathologically, of the teeth. The connecting link between the mechanical dentist on the one hand, and the physician on the other, has probably not until recently been sufficiently recognized. Works such as this have a tendency to give prominence to the fact that dentistry, in the best sense of that term, is more than a mechanical trade, and show how a liberal medical education best fits the dentist for his calling, and, having thus been educated, he is entitled to recognition as a specialist in the field of medicine.

This work can be commended alike to the mechanical dentist, the dental specialist, and the physician. The publisher has issued it in a form worthy of so deserving a work.

The Year-Book of Treatment. Philadelphia: Lea Brothers & Co. Chicago: A. C. McClurg & Co. Pp 344. 1889.

The preface to the book gives its object so fully that those not already familiar with its scope can ascertain it from that preface reproduced herewith: "The object of this book is to present to the practitioner not only a complete account of all the more important advances made in the Treatment of Disease, but to furnish also a review of the same by competent authorities.

"Each department of practice has been fully and concisely treated, and care has been taken to include such recent pathological and clinical work as bears directly upon Treatment.

"The medical literature of all countries has been placed under contribution, and the work deals with all the more important matters relating to Treatment that have been published during the year ending September 30th, 1888.

"A full reference has been given to every article noticed."

Text-Book of Medical Jurisprudence and Toxicology. By John J. Reese, M.D., Professor of Medical Jurisprudence and Toxicology in the University of Pennsylvania, etc., etc. Second Edition. Revised and Enlarged. Philadelphia: P. Blakiston, Son & Co. Chicago: W. T. Keener. Pp. xvi and 646. 1888.

The first edition of this work, which appeared about five years ago, has become familiar to the profession, and it only remains to note the fact that this edition has been carefully revised and some new matter added, including the subjects of Blood Stains, Suffocation, Ptomaines, Malpractice, and Toxicology. Whilst the first object of the book is to meet the requirements of students of Legal Medicine it will still be found useful and interesting to others.

In preparing this edition the author has sought to bring it up to the literature of the day, and his effort has not been unsuccessful. The publishers have issued it in good shape—worthy of so good a book.

Rectal and Anal Surgery, with Description of the Secret Methods of the Itinerant Specialists, by Edmund Andrews, M.D., LL.D., and Edward Wyllys Andrews, A.M., M.D., Professors of Clinical Surgery in Chicago Medical College, etc., etc. Second Edition, Revised and Enlarged, with Illustrations and Formulary. Chicago: W. T. Keener. pp. xiv. and 140. 1889.

When the first edition of this work appeared, so recently, so full a review of it was given in this journal, that it remains only to draw attention to the fact that a second edition has so soon been called for, and that the authors have taken advantage of the opportunity to improve upon the first edition by re-writing and enlarging much of the book. Several new chapters and an appendix have been added, and the number of illustrative cuts has been increased. In many respects this edition will be found to be an improvement on its predecessor. The mechanical execution of the work is creditable to the publisher.

Saint Bartholomew's Hospital Report. Edited by W. S. Church, M.D., and W. J. Walsham, F. R. C. S. Vol. xxiv. London: Smith, Elder & Co. Pp. xxii and 448. 1888. With Supplement giving Statistical Tables of the Patents under Treatment during the year 1887.

The volume contains twenty-nine articles on as many different subjects, with a list of specimens added to the Museum; Donations to the hospital library; List of Scholarships and Prizes; List of Prize-men; Hospital Staff, and a List of Subscribers to the hospital fund.

The text of the report is liberally illustrated, and the illustrations are fairly well executed.

The volume is fully up to the high standard of its predecessors, which have become so well known to the profession, and so justly esteemed.

A Reference Hand-Book of The Medical Sciences—Volume VII. Edited by Albert H. Buck, M.D. New York: William Wood & Company.

This excellent work has been made familiar to the medical profession by the six volumes that have preceded this one, which begins with Ter., and ends with Wor. Its main characteristics are similar to those of the other six volumes, including the illustrations which are quite numerous. The work reflects much credit on the editor and the publishers.

Lectures on the Errors of Refraction and Their Correction with Glasses, Delivered at the New York Post-Graduate Medical School, with Illustrative cases, etc. By Francis Valk, M.D., Lecturer on The Diseases of The Eye, New York Post-Graduate Medical School, etc., etc. New York and London: G. P. Putnam's Sons. Chicago: W. T. Keener. 1889.

The work embraces eleven lectures, covering the anatomy of the eye—Refraction, Emmetropia, Hypermetropia, Myopia, Ophthalmoscopy, Muscular Asthenopia, Astigmatism, Retinoscopy, Presbyopia, and concludes with histories of illustrative cases, and specimens of Snellen's Test-types.

It is liberally illustrated with fairly good woodcuts. It is printed on good paper, in clear type.

The Student's Text-Book of the Practice of Medicine. By Angel Money, M.D., London, Assistant Physician to University College Hospital, etc., etc. London: H. K. Lewis. pp. xiv. 458.

The author of this little volume says, in his preface, that "To give a right conception of disease and its treatment has been more the writer's aim than to state all that has been written about it." That aim, as all will admit, is a commendable one, and perhaps the author has succeeded as well as could be expected, in so limited a space.

It is divided into fifteen chapters; has a *Therapeutic Index;* a general index, and a list of the author's published writings.

